# Towards Ideal Health Ecosystem With Artificial Intelligence-Driven Medical Services in India: An Overview

**DOI:** 10.7759/cureus.48482

**Published:** 2023-11-08

**Authors:** Dimple Kumar, Abhishek Ingole, Sonali G Choudhari

**Affiliations:** 1 Epidemiology and Public Health, Jawaharlal Nehru Medical College, Datta Meghe Institute of Higher Education and Research, Wardha, IND; 2 Community Medicine, Jawaharlal Nehru Medical College, Datta Meghe Institute of Higher Education and Research, Wardha, IND

**Keywords:** cost-effectiveness, deep learning (dl), machine learning (ml), precision medicine, artificial intelligence (ai)

## Abstract

Artificial intelligence (AI) has immense power to set up an ideal health ecosystem through "intelligent medicine" i.e., a combination of human and machine intelligence. However, the application of AI in healthcare is still unclear. Currently, India is facing huge challenges such as the scarcity of medical resources and the uneven distribution of medical services. This also highlights the opportunities linked to challenges and risks. The most recent pandemic has accelerated this process by acknowledging that medicine stands on the brink of an AI revolution. Incorporating the evidence on the role of precision medicine, cost-effective healthcare, and expanding humanistic and medical services, this paper demonstrates the digital health interventions for the “enhancement” of capabilities, “efficiency,” “extension of services” and upgrading “experience” in the health sector. Through thorough literature searches from PubMed, Google Scholar, and other reliable sources, this study aims to understand the evolving needs, and greater control and to bridge gaps in access to healthcare through AI. Also, India is currently developing the potential to automate multiple tasks and calling for more human interventions. The future of AI in healthcare looks promising with digital health interventions that eventually offer flexibility and convenience to both the patient and the provider. This paper will help public health professionals address ethical considerations and policy-making where AI plays a significant role in setting up an ideal health ecosystem.

## Introduction and background

“Intelligent medicine” is the ideal "health ecosystem," which integrates cutting-edge digital and intelligent technologies across healthcare [1]. Digital technology has been a revolutionary foray into the fields of education, business, and research, and recently, a quiet revolution has taken place in the modern healthcare system [2]. Over the first two decades, the worldwide medical and healthcare domain has made significant advancements with the accelerating growth of the economy, science, and tech [1]. However, the medical analog is still in its infancy [3], also the global shortage of medical resources and the uneven distribution of regional medical services continue to be serious problems [1]. It is no longer sufficient to lower age-adjusted death rates. Instead, the question now is: Are we ready for what is about to come? Would this innovation, medicine, or payment strategy change? How satisfied or happy people are, how far they can run, or even how healthy they will be in the future in terms of retaining organ capacity? [2]. In addition, the lack of openness in the algorithms' decision-making processes has been a major critique of other uses of artificial intelligence (AI) in education [4]. Moreover, the pandemic has exposed long-standing system problems and marginalization of public health. While extensive community health and regulatory initiatives are necessary to address this situation, it's essential to see this as a chance to deliver more smart and effective care despite rising patient loads and decreasing resources [5].

Intelligent medicine will significantly advance world health in the following areas: A) Encouraging precision health management for individuals and the whole population; B) Elevating the cost-effectiveness of healthcare services; C) Improving the humanistic services, patient satisfaction, and experiences with healthcare services; D) Extending the time, space, and geographic scope of medical services [1]. AI also means a paradigm shift in the doctor-patient relationship, as the well-known doctor-patient hierarchy is replaced by a relationship at an equal level as a result of digital health [6].

The aim of this study is to better understand the evolving needs, and greater control and to bridge gaps in access to healthcare through AI. India is currently developing the potential to automate multiple tasks and expecting more human interventions, especially in health sectors, by taking initiatives such as accessible applications and mobile healthcare facilities in rural and urban areas. In addition, this paper will help public health professionals in policy-making where AI plays a significant role as a mode of quality healthcare delivery to set up an ideal health ecosystem.

## Review

Methodology

**Figure 1:**
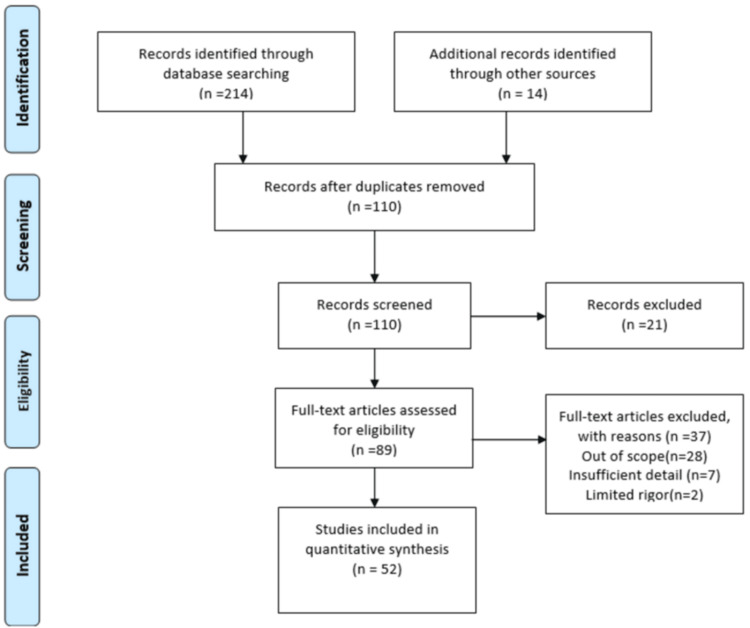
Inclusion and exclusion criteria of the study

This narrative review focuses on the role of AI as a mode of quality healthcare delivery. We searched PubMed, Google Scholar, and other sources such as the Ministry of Health and Family Welfare, Ministry of Commerce and Industry, Ministry of Electronics and Information Technology, National Health Portal of India, and Google to identify the relevant articles and reviewed publications using full-text search by using medical subject headings terms as well as we searched for key references from the relevant bibliography section. The review includes the various studies published from 2013 to 2022. The language of the study is English, and studies in other languages were excluded. Several keywords were used to find relevant articles that included the following: AI in healthcare, precision medicine, digital twins, cost-effectiveness, and mHealth. The selection of articles and their process are shown in Figure 1.

Discussion

Precision Healthcare

**Figure 2:**
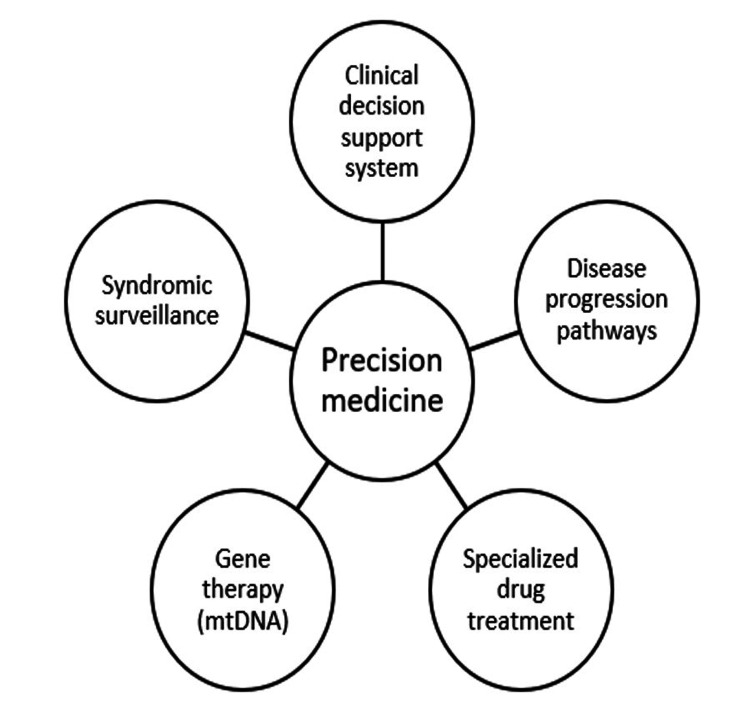
Advantages of precision healthcare mtDNA: Mitochondrial deoxyribonucleic acid

Precision healthcare involves data science techniques that have the potential to adapt early therapies for each patient [7,8]. Precision medicine, referred to as “personalized medicine,” is a cutting-edge method to customize and enhance the prognosis, treatments, diagnosis, and prevention of any disease through the usage of massive and complex datasets. Based on readily available multidimensional clinical and biological datasets, high-performance computing and AI can take into account the unique differences in clinical characteristics, including genes, proteins, metabolites, environment, and lifestyle and can also predict disease risk more accurately [7,9]. As precision approaches advance, they must also support the Internet of Things (IoT) [10], as well as the ethical and transparent use of data [11]. Figure 2 shows the various advantages that precision healthcare offers [7,9,12].

In this perspective, AI consists of machine learning (ML) and deep learning (DL) [7].

ML: It is a statistical method for "learning" through "teaching" models with data and fitting models to data. Precision medicine is one of the basic traditional ML techniques, that determines which treatment procedures are likely to be effective on a patient, based on a variety of patient traits and the context of the therapy [13,14].

DL: A crucial step in the process of drug discovery is predicting the binding affinity and binding interaction strength between the ligand and the target protein. DL applications in the area of medical research almost doubled in 2016 [15]. The ligand binds to the target protein using various virtual screening techniques, forming a protein-ligand complex that causes effects including protein inhibition and activation [16]. There are certain applications of ML and DL that are shown in Table 1 [3,13-17].

**Table 1:** Applications of ML and DL ML: Machine learning; DL: Deep learning; NLP: Natural language processing; AI: Artificial intelligence

ML	DL
NLP	eHealth
Neural networks	Drug design
mHealth	Drug discovery
AI devices	Visual data recognition
Diagnostic imaging	Speech recognition
Electro imaging	Simplifying clinical trials
Mass screening	Virtual learning
Emergency care	Digital twins

A key sector of the healthcare Information Technology market that has experienced recent rapid expansion is "mHealth."

mHealth: Health devices such as wearable sensors and smartphones outside of hospital grounds for remote in-home treatment have expanded in tandem with the rise in IoT device usage. These prediction models find new approaches to the detection and treatment of chronic illnesses and psychological issues by utilizing mHealth data. Hospitals and other healthcare facilities are using these devices more frequently to monitor patients continuously and to check on the capacity of intensive care units. A significant benefit of using DL in the field of mHealth analytics is the creation of strong algorithms, which facilitate the provision of preventive medicine and care to vulnerable individuals [18].

Cost-Effective Healthcare Services

It is an open invitation to collaborate fruitfully with the AI sector. Facilitating innovation inside and around health systems like the National Health Service is a top concern for policymakers and clinical leaders due to the moral necessity for improvements in patient care by using a patient-centered approach and the requirement that these advances be cost-effective [19].

Health technology assessment (HTA): HTA is becoming more mindful of the necessity of using a patient-centered approach when identifying how best to manage the limited resources of time, money, and technology. HTAs focus mainly on the assessment of available data and deciding on new therapy financing. Clinical evidence and pharmacoeconomic studies, such as cost-effectiveness analyses, budget impact analyses, and/or cost-utility analyses, are usually required for HTAs. HTA bodies are allowed to decide the refund judgments by assessing this evidence [20].

**Figure 3:**
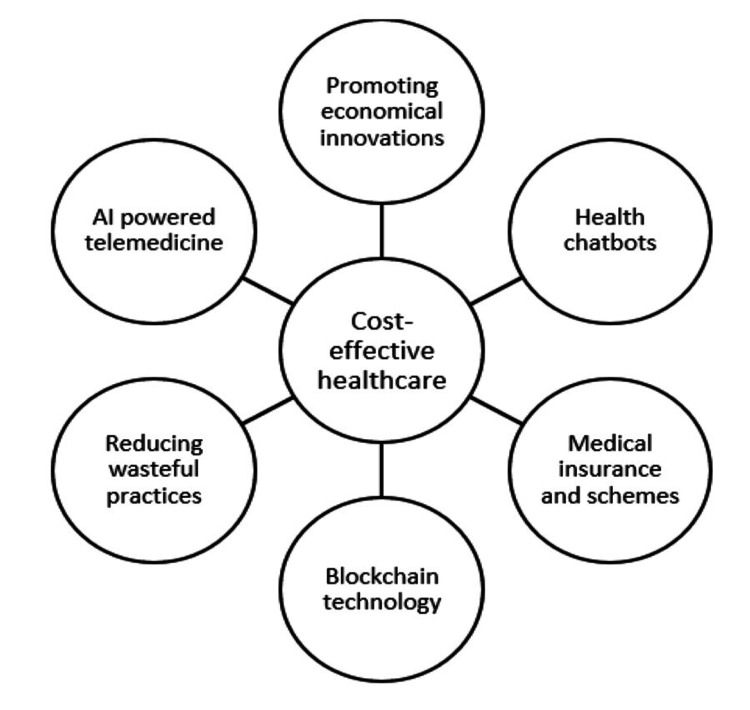
Ways to achieve cost-effectiveness AI: Artificial intelligence

Research in this area has been continually expanding, and in various studies, the cost-effectiveness of digital healthcare for type 2 diabetes and hypertension [21], mental health [22], as well as telemedicine for distant orthopedic consultations [23], atrial fibrillation [24], hospital-acquired pressure injuries [25], colorectal cancer [26], Food safety risk management [27] was thoroughly examined. The healthcare provider, insurance, pharmaceutical, and medical technology industries stand to benefit the most from it [28]. AI chatbots and voice bots are also identified as significantly aiding doctors and cutting healthcare costs [29]. Also, there are other ways for advancing cost-effective healthcare as shown in Figure 3 [5,30,31].

Enhancing Humanistic Services and Patient Experiences

So far, the primary causes of the healthcare workforce crisis are the scarcity of doctors globally, the aging and burnout of physicians, as well as increased demand for chronic care. The key components of an efficient system are the availability, accessibility, acceptability, and caliber of its health professionals. AI shows potential for filling these gaps [6]. Digital scribes have the potential to ease the strain of manual documentation. The redesign of the clinical encounter will be the cost of this assistance [32]. But India now has a space to enhance smart humanistic services and patient satisfaction as described in Table 2 [1,2,29,33].

**Table 2:** AI-driven services to enhance humanistic services and patient satisfaction AI: Artificial intelligence

Enhancing humanistic services	Enhancing patients' experiences
Measuring health status	Equal healthcare access
24/7 access to healthcare	Faster services
Health education to promote wise choices	Greater accuracy of digital devices
Provide enough fee-for-service to physicians	Triage services
Emergency care management	Medical expert systems (mHealth)
Addressing social determinants of health	High-performance medicines
Eliminate the effect of geography	Beneficial schemes/insurance
Update about the appropriateness of care	Exploration of patients’ expectations

Extending Medical Services

**Figure 4:**
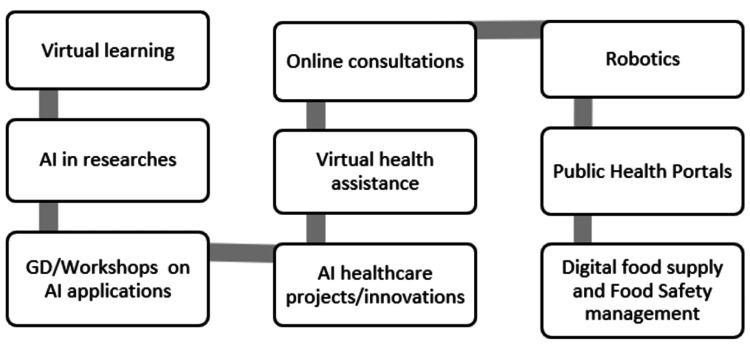
Methods to extend healthcare services AI: Artificial intelligence; GD: Group discussion

AI is gradually changing medical practice. There are various AI applications in medicine that can be employed in a number of medical domains, such as clinical, diagnostic, rehabilitative, surgical, and prognostic techniques [34]. With new advancements in AI poised to dramatically alter medical practices, interest in training present and future physicians on AI is growing. With this interest arises the question of what, specifically, medical students should learn and how they can make medical services convenient for the patient in any geographical region [35]. Figure 4 shows the digital methods to extend healthcare services in India [36-39].

Other Uses of AI

Implementation science: It is a relatively new topic, but is capable of significantly improving our understanding of AI implementation through the development of new theories, models, and frameworks. This versatile approach, merging AI and implementation science, surpasses the conventional limits of each of the sciences. Pilot studies and feasibility studies are necessary components in the way to implementation. According to Curran et al., efficacy studies come first, then effectiveness studies, and finally implementation research [40]. Most studies aid in creating a shared understanding of AI in the development of AI-enhanced surgical teaching tools, such as virtual simulations that provide tailored, adaptable, and dynamic learning settings that are not otherwise possible, are currently progressing [41].

Digital twins: Digital twins, personalized simulation models that were first used in business, are now being used in healthcare and medicine, with some outstanding results, such as in the immune system, control of insulin pumps, and cardiovascular diagnostics. Because the immune system is essential in a wide range of illnesses and medical ailments, from battling viruses to autoimmune disorders; therefore, it will have a particularly large impact [3].

Robotics

To reduce infections: Robotics can be applied in infectious disease outbreaks to reduce additional exposure through robotic disinfection, drug and food delivery, vital sign monitoring, and border control [42], automatic chronic disease detection, real-time suicide prediction and intervention, aiding emergency response, enabling patient rehabilitation, offering noninvasive care, and avoiding medical mistakes are just a few examples [18].

Comrade robot: Its primary duty is to sense surroundings through hearing, seeing, and touching. Gathering information including clinical findings, a client's medical records, and prior medical incidents can offer advice on preventive and prognosis. It is believed that the robot will help doctors identify the origin of the patient's symptoms more quickly and reduce errors [43].

Surgical robots: They give surgeons "superpowers," enhancing their vision, and capacity to make precise, minimally invasive incisions, close wounds, and other surgical procedures. They were first approved in the USA in 2000. Yet, important choices are still made by human surgeons. Gynecologic surgery, prostate surgery, and head and neck surgery are among the common surgical procedures performed with robotic surgery [13].

AI-Healthcare in India

When considering the possibilities of AI and health, it was discovered that the various segments use AI in a number of ways as shown in Table 3 and to strategize its approach, India is taking many initiatives as shown in Table 4.

**Table 3:** Uses of AI tools in different Healthcare sectors of India Note: Usually, descriptive and predictive AI is used in all healthcare sectors [44]. AI: Artificial intelligence; ML: Machine learning; CRM: Customer relationship management; CV: Cardiovascular; TB: Tuberculosis; MINE: Microsoft Intelligent Network for Eyecare; MOM: Mobile Obstetrics Monitoring

Healthcare sectors	Uses	AI tools
Hospitals (government hospitals and private hospitals)	Treatment of disease; data analysis and research evidence; improving quality of care	Microsoft Azure, ML, data analytics, CRM online, and Office 365 to improve patient care; and treatment of different forms of cancers, and diabetes-related diseases.
Pharmaceuticals	Drug discovery; automation of pharmaceutical supply chain management; customizing marketing messaging for customer engagement	Apps for CV, liver, and vertigo diseases. Pharmarack software-as-a-service (SaaS) for pharmaceutical supply chain management
Diagnostics	Analytical or diagnostic services	Niramai (Non-Invasive Risk Assessment with Machine Intelligence) applies thermal analytics for early-stage breast cancer identification; Advenio Tecnosys identify TB; Qure.Ai for treatment plans; Wysa & Woebot chatbots for mental health support
Medical equipment and supplies	Manufacturing AI-based medical equipment and supplies; monitoring vital signs of patients in ICUs	Niramai - detection of early signs of breast cancer; wireless patch for heart patients that monitors vitals and transmits this data via the cloud; Consultative Critical Care that observes heart rates and automatically administers shocks in unusual cases.
Medical insurance	Health insurance and medical payment facilities, covering hospitalization expenses and use of Chatbots, voice-bots to interact with patients; analyzing considerable amounts of data within a while; identifying suspicious patterns in data	Boing & MyRA chatbot to address customer queries on motor and health insurance, to sell insurance policies; HDFC Life’s email bot Spok claims to be the first in India to automatically read, understand, categorize, prioritize, and respond to customer emails.
Telemedicine	Provide clinical services to patients for follow-up visits, management of chronic conditions, medication, specialist consultation, and other clinical services that can be provided remotely via secure video and audio connections	SigTuple - to generate a pathology report without assistance from a pathologist; MINE; MOM software to identify and treat high-risk pregnancies.

**Table 4:** Initiatives taken by the Government of India NITI: National Institution for Transforming India; IT: Information Technology; S&T: Science and Technology; UIDAI: Unique Identification Authority of India; DRDO: Defence Research and Development Organisation; NASSCOM: National Association of Software and Service Companies; IPR: Intellectual Property Rights; HDFC: Housing Development Finance Corporation

Initiatives/schemes	Proposed by/year	Description
National eHealth Authority (NeHA) [44]	Ministry of Health and Family Welfare in 2015	It aims to build an integrated health information system by partnering with various stakeholders. Planning the “National eHealth Policy and Strategy” FUNCTIONS: Lay out data, handling, privacy and security policies, guidelines, and health records of patients.
Artificial Intelligence Task Force [45]	Ministry of Commerce and Industry in 2017	It aims for India’s Economic Transformation through AI FUNCTION: Government organizations (NITI Aayog, Ministry of Electronics and IT, S&T, UIDAI, DRDO) and experts, academics, researchers, and industry leaders are working on Integrating AI in our Economic, Political and Legal thought processes.
Policy Group on Artificial Intelligence [44]	Ministry of Electronics and Information Technology	Its aim is to create a road map and policy framework for AI adoption. FUNCTION: The policy group comprises individuals from academia and NASSCOM for an industry viewpoint, and focuses on problems such as privacy, security, accountability, and skilling the workforce.
National Intellectual Property Rights Policy [44]	Department of Industrial Policy and Promotion (DIPP) in 2016	It aims to create awareness to strengthen the enforcement of IPR. FUNCTION: Recognizes the creative potential of new and emerging technologies for improved access to inexpensive healthcare solutions and pharmaceuticals.
United States–India Science & Technology Endowment Fund (USISTEF) [46].	Government of the US and India	It aims to support cooperative activities of the US-India that would lead to invention and entrepreneurship through science & tech. FUNCTION: The Fund chooses and helps economically sustainable joint US-India entrepreneurial projects.
Cognitive Science Research Initiative (CSRI) [47]	Department of Science and Technology	It aims to help the scientific community deal with issues related to social problems and cognitive impairments. FUNCTION: The CSRI offers funds to support innovative studies pertaining to cognitive science.
Biotechnology Ignition Grant Scheme (BIG) [48]	Biotechnology Industry Research Assistance Council (BIRAC) in 2012	It aims to focus on challenges faced by startups in the biotechnology and medical device sectors due to long biological time and unreliable markets. FUNCTION: Up to INR 5 million in funding will be given to the most inventive concepts to help them develop and reach proof-of-concept.

Challenges to AI in India

Data access: The barrier to implementing AI in healthcare is not technology, rather it is data access. Large medical datasets are difficult to access for research purposes for legal or other reasons. The two biggest issues with data are getting consent for the gathering and making sure the data is accurate and consistent [44].

Data safety and privacy: Keep in mind that the reliability and quality of healthcare data can vary. AI-based tools can be used by hackers to gather sensitive data, including electronic health records. Moreover, ML algorithms may be abused to create autonomous methods that compromise the security and safety of such crucial data, though the real-time collection and usage of a wide range of data may or may not be revealed to a patient with consent taken [44].

Adoption of AI due to algorithm bias: Algorithms continue to demonstrate their superiority in terms of diagnostic "accuracy." The "black box" nature of many of these ML techniques, however, makes it challenging to deduce any diagnostic reasoning. The difficulty in interpreting AI models poses a barrier to adoption, which is sensible given the possibility that these models could learn hidden biases from training datasets and display discrimination in their output without our knowledge [49].

Treatment challenges: Multidisciplinary research is impacted by the domain distinctions between AI and implementation science. Improving trial efficiency, better protocol, utilizing improved data sources, and demonstrating the importance of the results, overcome new treatment challenges [40].

Medico-legal context: Even under the current medical regulations, lines of responsibility are not always defined when medical errors occur, and it is even less clear where those obligations should lie when AI "bots" increasingly support or even offer healthcare services on their own [50]. In order to offer secure patient care, AI healthcare projects must be trusted [51].

Implementing the changes in education: One of the biggest obstacles for today's medical educators may be responding to those changes, in addition to providing the content and approach of specific teaching. As a result, it appears that training educators are essential for improving current methods and putting this growing body of advice into practice [52].

## Conclusions

Several studies in this article conclude that AI has the potential to revolutionize healthcare. In India, AI-powered solutions are playing a valuable tool in bridging the gap and improving access to quality healthcare. Precision medicine has focused on prevention and risk assessment by identifying individuals at high risk of developing certain diseases, proactive interventions and preventive measures can be taken to reduce disease progression. The use of AI-driven healthcare applications in India has led to the analysis of large amounts of patient data, identifying patterns, and making predictions about disease outcomes. Chatbots and virtual assistants have also enhanced patient care by providing immediate responses to common medical queries, scheduling appointments, and offering medication reminders. The future of AI in healthcare looks promising with continued advancements and integration into various aspects of the industry. Precision healthcare can assist health professionals in making more informed decisions, providing personalized treatment plans, and predicting patient outcomes. Automating multiple tasks, streamlining workflows, and reducing human error will ultimately save time and resources. Digital medical education also has many advantages in terms of accessibility, flexibility, interactive learning global collaboration, and cost-effectiveness. Our study suggests addressing the unwelcomed areas and challenges including ethical considerations ensuring patient privacy, data security as well as algorithm biases. This will undoubtedly advance the relationship between humans and machines. In the context of public health alertness and response during outbreaks or public health emergencies, there is a need to integrate AI, robotics, and telemedicine with an organizational structure powered by AI to speed up healthcare delivery and improve access to healthcare. Thoughtfully planned and executed digital health interventions and implementations will increase the benefits of AI in every way.
